# Peer-mentor support for older vulnerable myocardial infarction patients referred to cardiac rehabilitation: single-arm feasibility study

**DOI:** 10.1186/s40814-022-01141-w

**Published:** 2022-08-09

**Authors:** Maria Pedersen, Birgitte Bennich, Takyiwa Boateng, Anne Marie Beck, Kirstine Sibilitz, Ingelise Andersen, Dorthe Overgaard

**Affiliations:** 1 Department of Nursing and Nutrition, University College Copenhagen, Tagensvej 86, 2200 Copenhagen N, Denmark; 2 Department of Nursing and Nutrition, University College Copenhagen, Sigurdsgade 26, 2200 Copenhagen N, Denmark; 3 The Dietetic and Nutritional Research Unit, EFFECT, Herlev and Gentofte University Hospital, Borgmester Ib Juuls Vej 50, 2730 Herlev, Denmark; 4 Department of Cardiology, Rigshospitalet, The Heart Centre, Copenhagen University Hospital, Blegdamsvej 9, 2100 Copenhagen Ø, Denmark; 5 Section of Social Medicine, Department of Public Health, Faculty of Health and Medical Sciences, University of Copenhagen, Øster Farimagsgade 5, 1353 Copenhagen K, Denmark

**Keywords:** Mentors, Patient participation, Patient perspectives, Rehabilitation, Inequalities in health, Coronary heart disease, Self-efficacy, Quality of life, Lifestyle, Cardiothoracic nursing

## Abstract

**Background:**

The positive effects of cardiac rehabilitation are well established. However, it has an inherent challenge, namely the low attendance rate among older vulnerable patients, which illustrates the need for effective interventions. Peer mentoring is a low-cost intervention that has the potential to improve cardiac rehabilitation attendance and improve physical and psychological outcomes among older patients. The aim of this study was to test the feasibility and acceptability of a peer-mentor intervention among older vulnerable myocardial infarction patients referred to cardiac rehabilitation.

**Methods:**

The study was conducted as a single-arm feasibility study and designed as a mixed methods intervention study. Patients admitted to a university hospital in Denmark between September 2020 and December 2020 received a 24-week peer-mentor intervention. The feasibility of the intervention was evaluated based on five criteria by Orsmond and Cohn: (a) recruitment capability, (b) data-collection procedures, (c) intervention acceptability, (d) available resources, and (e) participant responses to the intervention. Data were collected through self-administrated questionnaires, closed-ended telephone interviews, semi-structured interviews, and document sheets.

**Results:**

Twenty patients were offered the peer-mentor intervention. The intervention proved feasible, with a low dropout rate and high acceptability. However, the original inclusion criteria only involved vulnerable women, and this proved not to be feasible, and were therefore revised to also include vulnerable male patients. Peer mentors (*n* = 17) were monitored during the intervention period, and the findings indicate that their mentoring role did not cause any harm. The peer-mentor intervention showed signs of effectiveness, as a high rate of cardiac rehabilitation attendance was achieved among patients. Quality of life also increased among patients. This was the case for emotional, physical, and global quality of life measures at 24-week follow-up.

**Conclusion:**

The peer-mentor intervention is a feasible and acceptable intervention that holds the potential to increase both cardiac rehabilitation attendance and quality of life in older vulnerable patients. This finding paves the way for peer-mentor interventions to be tested in randomized controlled trials, with a view toward reducing inequality in cardiac rehabilitation attendance. However, some of the original study procedures were not feasible, and as such was revised.

**Trial registration:**

The feasibility study was registered at ClinicalTrials.gov (ClinicalTrials.gov identification number: NCT04507529), August 11, 2020.

## Key messages regarding feasibility

- **What uncertainties existed regarding the feasibility?**

This feasibility study addresses several uncertainties, e.g., “Can we recruit appropriate participants?”; “How appropriate are the data-collection procedures and outcome measures for the study’s purpose and intended population?”; “Are the study procedures and intervention suitable for and acceptable to participants?”; “Does the research team have the resources and ability to manage the study and intervention?”; and “Does the intervention show promise in terms of successful outcomes among the intended population?”

- **What are the key feasibility findings?**

The intervention proved feasible, with a low dropout rate and a high level of acceptability. However, the initial inclusion criteria only included vulnerable female patients, and this proved not to be feasible in the clinical setting, and as such had to be revised to also include vulnerable male patients. The inclusion of peer mentors required that several sites had to ensure a large, eligible population of potential peer mentors to enable their timely inclusion. Data collection by self-administrated questionnaires proved feasible, albeit time-consuming, among the population of older vulnerable patients. The mixed methods intervention design required a diverse research team with quantitative, qualitative, and mixed methods qualifications. The peer-mentor intervention showed signs of effectiveness among the population of older vulnerable patients, as shown by the high rate of cardiac rehabilitation attendance and the increased quality of life, as measured at 24-week follow-up.

- **What are the implications of the feasibility findings for the design of the main study?**

The implications for the design of the subsequent randomized controlled trial (RCT) are that challenges associated with the recruitment of both patients and peer mentors must be taken into consideration. For this purpose, inclusion criteria were revised, and strategies for improving inclusion developed. The peer-mentor intervention, mixed methods intervention design, and data-collection methods are feasible and therefore could be incorporated into future peer-mentor intervention trials.

## Background

Cardiovascular disease is one of the main causes of morbidity, mortality, and hospitalization worldwide. Advances in treatment regimens have reduced cardiovascular mortality, resulting in an aging myocardial infarction (MI) population in need of cardiac rehabilitation (CR) [1]. The positive effects of CR are well established — it has proven effective in reducing cardiovascular mortality, lowering hospital admissions, and improving quality of life among patients with ischemic heart disease [1]. These positive effects have also been established among older patients [2]. However, there is an inherent problem, namely the low attendance rate, which is often below 50% [3]. Several studies have shown that low participation in CR (defined as both nonattendance and dropout) is most prevalent among older, female, and vulnerable patients [3, 4]. The term “vulnerable” covers patients with low socioeconomic position (SEP), defined as low educational level, patients with a non-Western background, and patients living alone, as all of these groups are characterized by particularly low CR attendance [3, 4]. Inequality in CR attendance is a known problem in several parts of the world, including the USA, the UK, and Europe [4]. Feasible interventions aimed at increasing CR attendance among these vulnerable groups are therefore warranted. However, older vulnerable patients are often underrepresented in cardiovascular research with a focus on improving CR [1].

Previous research has shown that older vulnerable MI patients often use social support to navigate both the disease trajectory and the healthcare system [5]. This group of patients especially values social support from peers, who are able to relate to the patients in ways that, e.g., professionals or relatives cannot [5, 6]. Peer mentoring (i.e., mentoring someone with a similar life situation or health problem as oneself) [7] is a low-cost intervention that holds the potential to improve CR attendance and physical and psychological outcomes among older patients. Previous research has shown that the peer-mentor method is effective in improving physical and psychological outcomes among patients with cardiovascular disease [6, 8]. The method has shown significant improvements in the fitness of older people and also reduces symptoms of depression [7]. The potential of peer-to-peer support is evident; to our knowledge, however, peer support has not been explored in a CR setting among older vulnerable MI patients.

The present study is part of the ongoing research program “Heartened” (in Danish: “HjertensGlad”). The findings will be used to develop a subsequent randomized controlled trial (RCT) aimed at testing the effects of peer-mentor intervention in terms of increased CR attendance and improvements in lifestyle (diet, physical activity) and psychological factors (anxiety, depression, quality of life, self-efficacy) among older vulnerable patients with MI. To our knowledge, no data exists regarding the feasibility of a peer-mentor intervention among older vulnerable MI patients in a CR setting.

## Methods

### Aim

The aim of the study was to test the feasibility and acceptability of a peer-mentor intervention among older vulnerable MI patients referred to cardiac rehabilitation. The findings therefore pave the way for future randomized controlled trials of peer-mentor interventions.

### Design

**Fig. 1:**
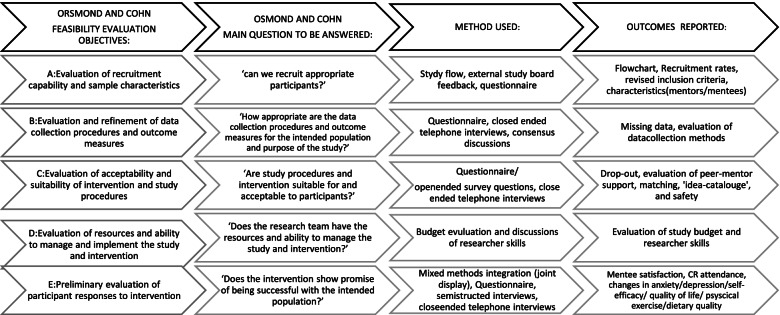
Feasibility study outcomes. Overview of feasibility methods and outcomes, guided by Orsmond and Cohns (2015). The distinctive features of a feasibility study: objectives and guiding questions

The study was conducted as a single-arm feasibility study and designed as a mixed methods intervention study. A convergent core design was used, and thus, quantitative and qualitative data were collected within the same timeframe to enable a more comprehensive understanding of study feasibility and acceptability [9]. The feasibility study was evaluated utilizing five (A–E) guiding questions proposed by Orsmond and Cohn [10] (see Fig. 1). The study was reported in accordance with the CONSORT extension to the guidelines for pilot and feasibility trials [11].

### External study boards

The study recruited a patient and public involvement board (PPI board) comprising four post-MI patients who were invited to contribute to the development and continuous refinement of the study, to ensure that it was designed and adjusted to reflect the patients’ preferences. Likewise, an advisory board of three external professionals with expertise in cardiac rehabilitation, interventional research, and social inequality was established, to enhance scientific quality and the study’s clinical relevance.

### Sample/participants

#### Inclusion of mentees

Patients (hereafter referred to as “mentees”) admitted to a university hospital in Denmark between September 2020 and December 2020 were invited to participate in the study. The hospital’s two cardiology departments (Hillerød and Frederikssund) are located 21 km apart, with a combined catchment area covering eight municipalities and 310,000 citizens. In Denmark, CR is offered as either a community-based rehabilitation program by the municipalities, a hospital-based interdisciplinary outpatient program, or a combination of the two. Research nurses at the hospital recruited the mentees and informed them about the study, orally and in writing. The research nurses screened the cardiology departments on a daily basis for eligible mentees and identified those who met the original inclusion criteria (see Table 1). Exclusion criteria: Those unable to provide written consent. The total number of included mentees was based upon previous research [3, 12].

**Table 1:** Inclusion criteria

	Original inclusion criteria	Revised inclusion criteria
Primary (all mandatory)	Female, *AND* ≥ 65 years, *AND* diagnosed with MI, *AND* referred to cardiac rehabilitation	≥ 65 years, *AND* diagnosed with MI, *AND* referred to cardiac rehabilitation
Secondary (one mandatory)	Low socioeconomic position^a^, *OR* lone dwelling, *OR* non-Western background^b^	Female, *OR* low socioeconomic position^a^, *OR* lone dwelling, *OR* non-western background^b^

^a^Defined as vocational educational level or below

^b^Defined as persons not born in Western Europe or North America

#### Inclusion of peer mentors

The researchers and research nurses recruited peer mentors via several locations, e.g., the university hospital, researchers’ network, or local departments at the Danish Heart Foundation. Inclusion criteria: both male and female patients diagnosed with an MI or a similar disease trajectory, e.g., percutaneous coronary intervention/coronary artery bypass grafting or chronic ischemic heart disease (no duration limit); and had previously attended the CR program (to serve as a positive role model). Exclusion criteria: Those unable to provide written informed consent.

### Peer-mentor intervention

The mentees were matched with peer mentors based on the individual mentee’s preferences in terms of, e.g., age, gender, geographical distance, and/or other factors expressed prior to the intervention. Throughout the intervention period (24 weeks), the mentee and the mentor were encouraged to have contact approximately eight times (no maximum was specified), depending on the needs of the individual patient. The number of recommended contacts was based upon the median number of contacts in a similar study population of older, economically disadvantaged patients with heart failure [13]. Mentor-mentee contact could take place via, e.g., telephone, email, face-to-face meetings, or other media. As a resource for the mentor-mentee meetings, the research group developed two inspiration catalogues (hereafter referred to as idea catalogues) — one with ideas for where face-to-face meetings might be held in local communities, e.g., a walk in a local park, and one with ideas for online meetings (due to the COVID-19 situation), e.g., preparing a meal together online. All peer-mentors were instructed to comply with current COVID-19 restrictions.

Peer mentors worked as volunteers and offered support and guidance based upon their own experiences of disease. They received training in the form of a 2-day online course organized by the study researchers and a psychologist specializing in peer mentoring. During the course, the peer mentors were introduced to the study concepts and received professional training in skills such as forming relationships with a focus on establishing trust, understanding psychological reactions to an MI and/or lifestyle changes, and training in communication skills (using open-ended questions and active listening).

All peer mentors were offered supervision by the research team and a psychologist appointed to the study and invited to attend online network meetings every second month during the intervention, as previous research has shown that supervision is an essential part of peer mentoring [8]. At the network meetings, peer-mentors were encouraged to share their experiences as peer mentors and inspire each other. Peer mentors were encouraged to contact the project leader if problems arose, e.g., with mentor-mentee contact.

### Data collection

**Fig. 2:**
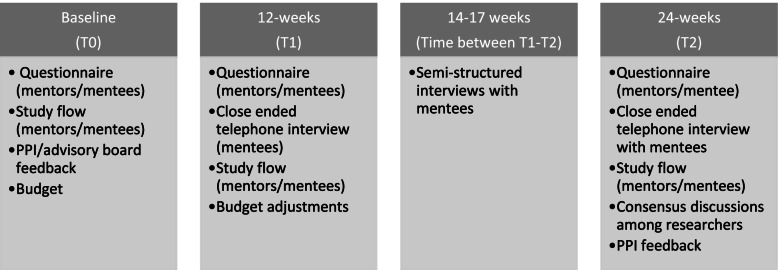
Data-collection time points. Overview of data collected at baseline (T0), 12 weeks (T1), 14–17 weeks (time between T1 and T2), and 24 weeks (T2)

The first author (MP) collected quantitative data among peer mentors and mentees, while the third author (TB) handled data management. Quantitative data were collected at three time points between September 2020 and June 2021: baseline (T0), 12 weeks (T1), and 24 weeks (T2) (see Fig. 2). Qualitative data were collected among a convenience sample of mentees who finished T1 data collection between mid-February and mid-March 2021, corresponding to weeks 14–17 post-inclusion (see Fig. 2). The second author (BB) collected and managed the qualitative data.

To evaluate the feasibility and acceptability of the peer-mentor intervention, quantitative and qualitative data were collected to address the five (A–E) guiding questions recommended by Orsmond and Cohn [10], as specified in Fig. 1. The data-collection methods were based on these five guiding questions (A–E).

#### Evaluation of recruitment capability and sample characteristics (A)

To evaluate recruitment capability and sample characteristics, and answer the guiding question “Can we recruit appropriate participants?”, we collected data on study flow and inclusion rate. Based upon findings in a similar population, a target inclusion rate of one patient every 3rd day was considered a success [3]. Data regarding sample characteristics for peer-mentors and mentees were collected through a self-administrated questionnaire capturing personal data (e.g., age, gender, education, cohabitation, country of origin). We also used verbal feedback from the PPI and advisory boards to evaluate and refine the inclusion criteria.

#### Evaluation and refinement of data-collection procedures and outcome measures (B)

To evaluate and refine the data-collection procedures and outcome measures, and answer the guiding question: “How appropriate are the data collection procedures and outcome measures for the intended population and purpose of the study?”, we tested the data-collection procedures to be used in the subsequent RCT study. The purpose was to assess if peer mentors and mentees were able to comply with and use the intended data-collection methods. Closed-ended telephone interviews were used to collect data relating to the primary outcome — self-reported cardiac rehabilitation (CR) attendance. Self-administered questionnaires (the psychometric properties of which are reported under section E) were used to collect data relating to the secondary outcomes (anxiety/depression, self-efficacy, quality of life, dietary quality, and physical activity). Several methods of questionnaire delivery were tested, including via mail order, online questionnaires, and personal delivery. The threshold for accepted missing values in questionnaires was informed by experience with dietary food-frequency questionnaires, which typically have the most missing values. As there are no guidelines regarding missing values in dietary data, the threshold for missing variables was a priori set at 30%, corresponding to the expected level found in other studies [14, 15].

To test the feasibility of qualitative data collection for the upcoming RCT, the first five mentees to finish the quantitative T1 data collection were invited to participate in a subsequent interview. Due to the COVID-19 situation, semi-structured telephone interviews were used to collect the qualitative data.

#### Evaluation of acceptability and suitability of intervention and study procedures (C)

To evaluate the acceptability and suitability of intervention and study procedures, and answer the guiding question “Are study procedures and intervention suitable for and acceptable to participants?”, we collected data on dropout rates among peer mentors and mentees. In the power calculation for the number needed to be included in the upcoming randomized controlled trial (*n* = 140), a dropout rate of 20% was recorded, based on results from a meta-analysis of attrition rates [16]. As such, in the feasibility study, dropout below 20% was considered a success. Additionally, peer mentors kept a record of the frequency, type, and topics of the mentor-mentee contacts, and their use of the idea catalogues when planning mentor-mentee meetings. The first author collected data regarding mentee preferences for peer-mentor matches (in terms of, e.g., age, gender, place of residence). As matching was considered an important aspect of the intervention, the team strived for a 100% match with patient preferences as a feasibility criterion. The first author performed and documented the mentor-mentee matching.

In addition, self-reported outcomes were used to monitor the peer-mentors’ safety and wellbeing during the intervention period, to detect signs of psychological strain or declining self-care (e.g., symptoms of anxiety and depression, a reduction in quality of life or self-efficacy, or lifestyle changes). Self-administered questionnaires completed by peer-mentors at baseline, T1 and T2 were used to collect data regarding anxiety/depression, self-efficacy, quality of life, dietary quality, and physical activity, to assess potential negative impacts of their mentoring role (the scales’ psychometric properties are described in section E). The first author kept records of the peer-mentors’ supervision needs.

#### Evaluation of resources and ability to manage and implement the study and intervention (D)

To answer the guiding question “Does the research team have the resources and ability to manage the study and intervention?”, we collected data regarding the budget and study expenses. Prior to commencement of the study, the research team discussed the skills already acquired to manage and implement the study; skills not covered by the research team were added to the budget in the form of salaries for external personnel.

#### Preliminary evaluation of participant responses to intervention (E)

To evaluate the participants’ (mentees’) responses to the intervention and answer the guiding question”‘Does the intervention show promise in terms of successful outcomes among the intended population?”; we collected both quantitative (closed-ended telephone interviews, self-administrated questionnaires) and qualitative (semi-structured interviews) data from mentees regarding both the primary and secondary outcomes that the upcoming RCT would use to evaluate the intervention. Supplementary data were also collected regarding the mentees’ satisfaction and the self-reported effects of the peer-mentor intervention.

Self-reported CR attendance (primary RCT outcome) was collected at T1 and T2 by closed-ended telephone interviews with mentees. Self-reported CR attendance has proven a valid method for collecting data regarding CR attendance [17]. CR attendance was defined on two levels: attended at least one CR session (physical training or dietary advice) or attended ≥ 50% of the physical training or dietary advice sessions. Based upon CR attendance among non-vulnerable groups [3], the success criterion was set at 50% CR attendance in the power calculation for the subsequent RCT.

The self-administered questionnaire consisted of the following scales:

Quality of life (QoL) was measured using the HeartQoL questionnaire [18] — a 14-item, disease-specific questionnaire with a global scale made up of physical and emotional subscales. The total possible scores range from 0 to 3 for each subscale, with higher scores indicating more positive outcomes. HeartQoL is a valid method for evaluating change in quality of life in patients with MI [18] and has also proven to be valid in Danish population studies [19].

Self-efficacy was measured using the general self-efficacy scale (GSE) [20]. The GSE scale consists of 10 questions. Possible scores range from 10 to 40, with higher scores indicating more positive outcomes. The scale is validated against patients recovering from myocardial events [21].

Symptoms of anxiety and depression were measured against the hospital anxiety and depression scale (HADS) [22], consisting of two subscales: HADS-A, for anxiety symptoms, and HADS-D, for depressive symptoms. Total possible scores range from 0 to 42, with lower scores indicating more positive outcomes. Scores above eight are indicative of the possible presence of anxiety/depression, while scores above 11 are indicative of probable presence of anxiety/depression. The scale is validated against a large sample of Danish patients with cardiac disease [23].

Dietary quality and physical activity were measured using the “HeartDiet” questionnaire [24]. Dietary quality was captured using a 19-item food-frequency questionnaire, consisting of two subscales: a fat score, with possible scores ranging from 0 to 100, and a fish-fruit-vegetable score ranging from 0 to 100. Higher scores indicate more positive outcomes. For the participants’ diet to be categorized as “heart healthy,” a minimum of 75 points must be achieved in both subscales. The questionnaire is validated against a Danish population of patients with cardiac disease [25]. Physical activity was measured based on number of times per week that participants spent at least 30 min exercising (e.g., brisk walking, running, cycling, swimming), and the results are categorized into four groups, based on the World Health Organization (WHO) and the Danish National Board of Health’s recommendations for weekly physical activity.

### Interview guide

The investigators devised a semi-structured interview guide, based on existing literature and theories [4, 5], covering themes corresponding to the quantitative data collected, i.e., acceptability of peer-mentor intervention, cardiac rehabilitation, quality of life, self-efficacy, anxiety and depression, diet, and physical activity. The quantitative questionnaires therefore provided the framework for the interview guide. However, all of the questions were rephrased into fewer and broader open-ended questions, where we invited the participants to openly communicate their views and experiences regarding CR and the implication of peer-mentor support [9]. To encourage a candid approach, the interviewers were unaware of the individual participants’ medical data and responses to the questionnaires. Initial ideas for data analysis, including with regard to the patterns and features of each participant’s responses, were recorded in field notes immediately after each interview [26].

### Data analysis

#### Statistical analysis

The quantitative data collected from peer mentors and mentees was subject to statistical analysis. Due to the small sample size (*n* = 20), a normal distribution of variables could not be firmly established. Descriptive statistics are therefore presented as median and 25–75% quartiles (Q1–Q3) for continuous variables. Categorical variables are presented as frequency distributions (numbers and percentages). All statistical analysis was carried out using IBM SPSS Statistics (Statistical Package for the Social Sciences, version 25).

#### Qualitative analysis

**Fig. 3:**
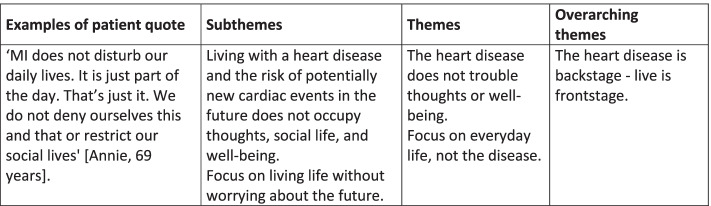
Example of theme development. Illustration of how overarching themes were developed from themes, subthemes, and examples of patient quotes

Braun and Clarke’s [26] six stages of thematic analysis were used to iteratively analyze, categorize, and interpret the mentees’ interview data:

1. Familiarization with the data by listening to the audio and noting initial interpretations and patterns. The first stage supported the following coding and analysis process, enabling an iterative process between different levels of analysis, and preserving the grounding in the raw data.
2. Generating initial codes based on the research question. The audio data were coded deductively, using preset codes informed by the notes from the first stage, but also inductively, via open-coding based on the data.
3. Searching for themes by collating corresponding codes into initial subthemes.
4. Reviewing themes by relistening to the audio data and developing preliminary broader themes. The initial field notes and notes from the first stage of the analysis supported this process.
5. Defining the “essence” of each theme while developing the main themes.
6. Writing up analytical narratives with illustrative quotes; see Fig. 3.

The analytic qualitative data analysis software NVivo (version 12 Pro) was used to support the organization of data. All names were altered to ensure anonymization.

Peer-mentor document sheets with notes regarding the content of mentor-mentee meetings were summarized into main conversation topics.

#### Mixed methods analysis

The mixed methods analysis consisted of merging quantitative and qualitative results in side-by-side joint displays (i.e., visual presentations integrating both quantitative and qualitative findings), which enabled a more comprehensive and nuanced understanding of the intervention’s feasibility and potential effectiveness [27]. The qualitative analysis was used to draw up analytical narratives with accompanying quotes, which were integrated into the joint displays along with the quantitative data. Mixed methods inferences (meta-inferences) were used to assess the fit between quantitative and qualitative findings. This resulted in either confirmation (findings reinforced each other), expansion (findings expanded insights), or discordance (findings contradicted each other) [27].

## Results/findings

The feasibility study was evaluated utilizing five guiding questions for evaluating a feasibility study, as recommended by Orsmond and Cohn [10]. The findings are reported on the basis of the five guiding questions (A–E).

### Evaluation of sample characteristics and recruitment capability (A)

#### Characteristics of mentees and peer-mentors

Table 2 shows the characteristics of the included mentees and peer mentors. The gender distribution was 60% male mentees and 80% male mentors. The median age was 76.8 for mentees and 66.5 for mentors. A total of 70% of mentees cohabited; this was the case for 79% of the mentors. All mentors were ethnic Danes. Of the mentees, 10% originated from other Scandinavian countries. The majority of mentees had vocational education (60%) or below (20%), while the majority of mentors (69%) had a short/medium/long-cycle higher education.

**Table 2:** Characteristics of included mentees and peer mentors

Variable	Level of variable	Mentees *n* = 20 *N* (%), median (Q1–Q3)	Peer-mentors *n* = 20 *N* (%), median (Q1–Q3)
Gender	Male	12 (60)	16 (80)
	Female	8 (40)	4 (20)
Age		76.8 (72.3–80.8)	66.5 (63.3–72.5)
Cohabitation	Cohabiting	14 (70)	15 (79)^a^
	Lone dwelling	6 (30)	4 (21)
Place of birth	Born in Denmark	18 (90)	19 (100)^a^
	Born outside Denmark (Scandinavian countries)	2 (10)	0 (0)
Education	7 or less years of education	3 (15)	1 (5)^a^
	10–11 years of education	1 (5)	1 (5)
	Vocational	12 (60)	4 (21)
	Short-cycle higher education	3 (15)	5 (27)
	Medium-cycle higher education	0 (0)	4 (21)
	Long-cycle higher education	1 (5)	4 (21)

*N* number, (%) percent, (Q1–Q3) first-third quartile

^a^Data regarding cohabitation, place of birth, and education is missing for one mentor, only full data on 19 mentors

#### Evaluation of mentees’ recruitment capability

Figure 2 shows the flowchart of included mentees. The inclusion process was initiated on 7 September 2020. However, no mentees were successfully included at this time. After an inclusion period of 45 days, only five eligible mentees could be identified, all of whom declined to participate in the study. Reasons to decline were lack of energy, interest, or time. The inclusion criteria were then reviewed. The feedback from the research nurses indicated that the inclusion criteria were too restrictive. Very few (*n* = 5) of the mentees met all of the inclusion criteria, and those who did were in a poor state of health that precluded their inclusion. Furthermore, the cardiology department experienced fewer MI patients during the COVID-19 pandemic, as has also been reported in other countries [28].

**Fig. 4:**
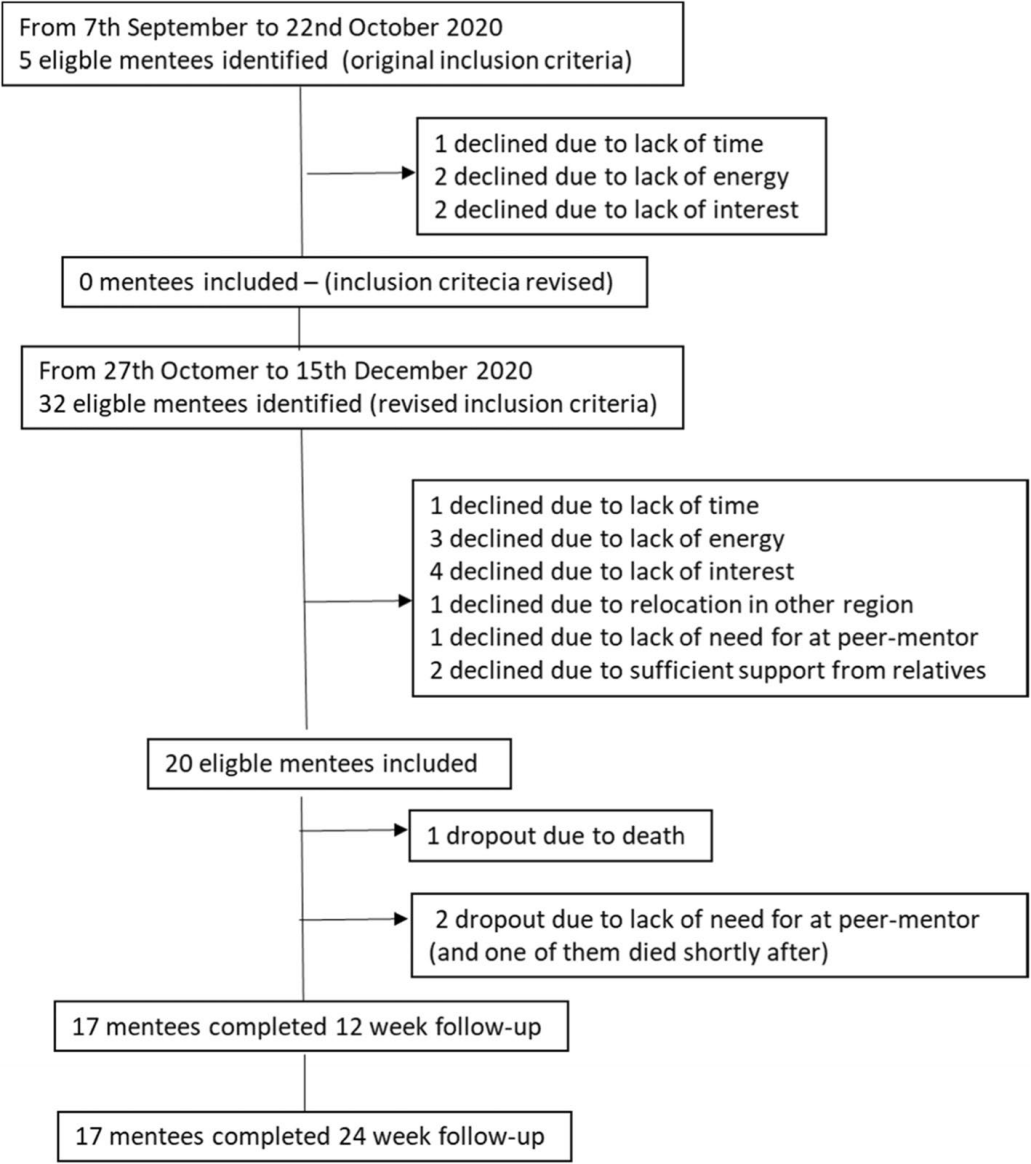
Flowchart of inclusion mentees. Flowchart of eligible mentees, number included, dropout (number and reasons), and number completed at 12- and 24-week follow-up

The inclusion criteria were revised after consulting the study advisory board and the PPI board. The inclusion process resumed on 27 October 2020 with new and revised criteria (see Table 1), in which the female gender was no longer a mandatory inclusion criterion (albeit remained a secondary one). This enabled male mentees to be included in the study. Over an inclusion period of 47 days, 32 eligible mentees were identified, 20 of whom agreed to be included in the study (see Fig. 4). This corresponds to an inclusion rate of one patient every 2.4 days. Figure 4 shows that three patients dropped out (15%) of the feasibility study, two did not feel the need for a peer-mentor, and one died before being matched with a mentor. As such, it was feasible to include patients at the estimated time.

#### Evaluation of peer-mentors’ recruitment capability

**Fig. 5:**
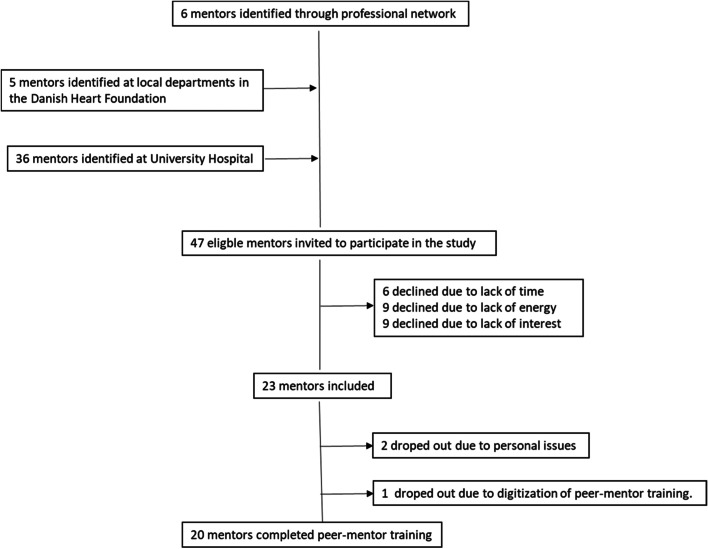
Flowchart of inclusion peer mentors. Flowchart of eligible peer mentors, number included, dropout (number and reasons), and number completing peer-mentor training

Figure 5 describes the peer-mentors’ inclusion in the study. Although the research nurses included mentors from the university hospital, the pandemic meant that nurses were restricted to COVID-19 duties and were unable to include mentors as planned. The research team therefore had to go elsewhere. Six mentors were recruited via the researchers’ network, and five were recruited through local departments at the Danish Heart Foundation. As of 7 September 2020, the project nurses were exempt from COVID-19 duties and could resume the inclusion of peer mentors from the cardiology department. The remaining peer-mentors were included over a period of 24 days (*N* = 36) (see Fig. 5). More than half (51%) of the eligible mentors declined to participate due to lack of time, low energy, or no interest. Eventually, 23 mentors agreed to participate in the study. Three (13%) subsequently dropped out due to personal issues or because of the COVID-19 restrictions (e.g., digitization of the peer-mentor training).

### Evaluation and refinement of data-collection procedures and outcome measures (B)

Data regarding self-reported CR attendance were collected through closed-ended telephone interviews. Self-reported CR attendance was achieved in 17/17 mentees (100%).

Self-administrated questionnaires were used to capture quantitative data among peer mentors and mentees. Among most peer mentors, it was possible to use an online version of the questionnaire, sent by e-mail. However, among mentees, several methods of delivery were considered. At first, questionnaires were sent by mail. However, this was not effective, as their return was considerably delayed, which hampered the implementation of the 12/24 week follow-up. Online questionnaires were also tested, but most mentees felt they lacked the IT literacy or confidence to use them. As a consequence, the team relied on personally delivering questionnaires to most mentees. This helped to ensure the successful return of questionnaires but also proved time-consuming. Nonetheless, this method resulted in the timely return of questionnaires in advance of the scheduled follow-up at 12 and 24 weeks. As such, the quantitative data collection method was viewed as acceptable and feasible. Due to COVID-19 restrictions, qualitative data were collected using semi-structured interviews by telephone.

Analysis of the baseline data revealed that most missing values occurred in the dietary data. The dietary fat variable was the most omitted in the pooled follow-up data, and full data was only achieved in 13/17 across all three time periods (T0/T1/T2). This corresponds to 23.5% missing data, which was deemed acceptable. However, the questionnaire was altered to increase correct completion of the dietary questionnaire by adding guiding text (“one tick per question”).

### Evaluation of acceptability and suitability of intervention and study procedures (C)

#### Evaluation of acceptability

In total, 3/20 mentees (15%) dropped out during the study period (see Fig. 4). The feasibility study revealed a large variation in support needs among mentees. The median number of contacts between mentee and peer mentor was eight (Q1–Q3: 5–10) (range: 1–12). Each contact lasted between a few minutes and several hours, depending on mentee needs. Most mentees (70%) received peer-mentor support for a period of 6 months. However, 30% of mentees unsubscribed from peer-mentor support earlier (1–3 months) due to lack of need. The variation in peer-mentor support needs among mentees was discussed with the PPI board. Their feedback resulted in a revised definition of the intervention that could accommodate the variation in needs. The intervention was therefore defined as peer-mentor support for a period of up to 6 months, with approximately eight contacts between peer mentor and mentee.

In total, 110 contacts were registered between peer mentor and mentee. Most mentor-mentee contacts were conducted via telephone (57%) or text message (26%), with 11% face to face and 6% via e-mail.

#### Evaluation of suitability

The peer mentors did not directly use the two idea catalogues in the planning of mentor-mentee meetings. Many peer mentors were already members of the local community and so planned face-to-face meetings at the patients’ homes or at a local forest or beach.

Table 3 shows how mentor-mentee matches were made. In 16 cases, mentees were matched with a same-sex mentor, and 14 matches were made with a mentor within the same age span. In 16 cases, it was possible to match a mentor and mentee who lived within a < 30-min commute from each other. Some mentor-mentee matches were based on similarities in terms of cohabitation, occupation, smoking history, medical history, labor-market activity, place of birth, education, or physical activity levels. All mentors were matched according to mentee preferences; however, some mentees had no preferences regarding the peer mentor with whom they were matched. Some preferred a mentor of the same sex or within the same age span. Some requested a mentor who lived nearby, while one requested a mentor with a similar medical history — specifically, someone who also had a pacemaker. As such, the mentor corps were regarded as successfully composed, with sufficient diversity.

**Table 3:** Matching criteria used

Gender	*n* = 16
Age (± 10 years)	*n* = 14
Place of residence (< 30 min commute from peer-mentors home)	*n* = 16
*Matches were primarily based upon the three abovementioned characteristics; however, below mentioned characteristics were also applied when relevant.*	
Cohabitation	*n* = 3
Past/present occupation	*n* = 3
Smoking (e.g., former smoker matchet with a smoker)	*n* = 2
Medical history (e.g., both have a pacemaker implanted)	*n* = 2
Labor market active	*n* = 1
Place of birth (e.g., both born in Norway)	*n* = 1
Education (e.g., both have long-cycle higher education)	*n* = 1
Physical activity level (e.g., both have a high physical activity level)	*n* = 1

Matching criteria used when matching mentee with peer-mentor. *N* = 19 (one mentee withdrew prior to match)

Mentor-mentee conversations were characterized by 11 main topics:

- Cardiac rehabilitation (motivation, trajectory, “mentees not receiving timely summons”)
- Physical activity and diet
- Heart disease and disease trajectory
- Hospitalizations/outpatient hospital visits
- Psychological influence of MI
- Everyday life
- Life stories
- Concerns about relatives (e.g., disease)
- The Danish Heart Foundation
- Medicine
- COVID-19 and vaccination

#### Evaluation of peer-mentor safety

To monitor peer-mentor safety and well-being during the intervention and ensure that no harm was inflicted due to their mentoring role, outcomes were also monitored among the peer mentors (data not shown). The overall data indicated no change during the study period (baseline — 24 weeks). However, at the 12-week follow-up, one mentor exhibited possible anxiety symptoms, and two others showed possible depression symptoms. The first author (MP) contacted these peer mentors by phone and/or e-mail to ensure their well-being. The follow-up revealed that their psychological symptoms were not due to the peer-mentor role but were caused by work-related stress. At the 24-week follow-up, no mentors showed signs of possible anxiety or depression symptoms. As such, there was no indication that peer mentors were being harmed by their role.

#### Supervision needs

During the intervention period, five mentors contacted the project leader because they had trouble establishing contact with their mentee (e.g., the mentee did not answer telephone calls or text messages). These issues were most often solved by the project leader facilitating the first contact between peer-mentor and mentee. Most peer mentors attended at least one of the network meetings offered every second month, and some attended all meetings. No peer mentors required individual consultations with the psychologist.

### Evaluation of resources and ability to manage and implement the study and intervention (D)

#### Evaluation of resources needed

The feasibility study had a budget of approximately £98,000/US $134,000. This covered salaries for the research team, project nurse, and psychologist (approximately 1700 working hours in total) and expenses associated with the questionnaire license. The mentors worked as volunteers, but the budget did include expenses for their training/supervision and travel expenses. The budget was exceeded in terms of expenses for peer-mentor training, since the peer mentors were recruited at different time points. As a consequence, the research team had to complete the peer-mentor training program several times. Due to the COVID-19 pandemic, all activities, e.g., network meetings and peer-mentor training, had to be adapted to an online setting, which increased working hours and costs for the research team. The feasibility study period also had to be extended due to the pandemic and difficulties in including patients who met the original inclusion criteria. This further added to the expenses and resulted in the budget and study process being revised during the study.

#### Evaluation of ability to manage and implement the study

The research team had the necessary skills to conduct the feasibility study. Skills not covered by the research team were obtained from external partners, e.g., a psychologist and a statistician.

### Preliminary evaluation of participant responses to intervention (E)

#### Qualitative findings

Semi-structured telephone interviews (*n* = 5) were conducted, with a duration between 12 and 55 min. Of the five mentees involved, two were female and three were male, with a median age of 78 years (range 69–80), 40% were lone dwelling, all were ethnic Danes, and 80% had lower education (vocational education or below).

#### Mixed methods findings

Table 4 shows both mentee satisfaction and the experienced effect of the peer-mentor intervention. In general, mentees reported being very satisfied (47%) or satisfied (41%) with the peer-mentor intervention at 24-week follow-up. A large proportion (70%) experienced a positive effect of the peer-mentor intervention. The qualitative data expand on this by indicating reciprocity in the mentor-mentee relation, i.e., the mentees did not experience the mentor-mentee relationship as highly unequal.

**Table 4:**
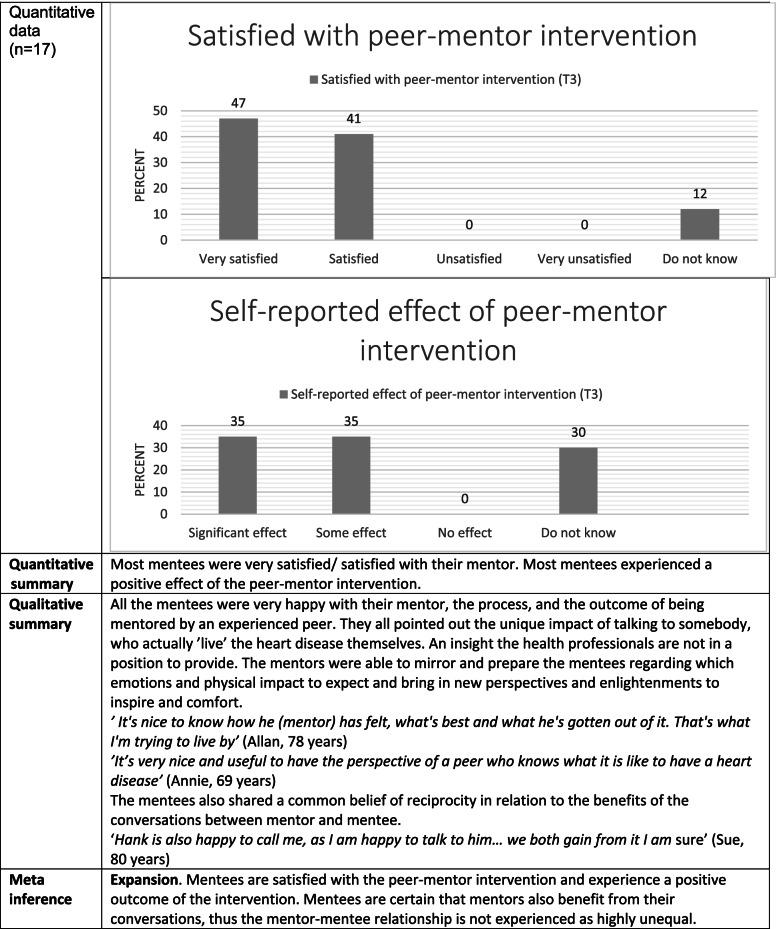
Joint display regarding mentee satisfaction and experienced effect of peer-mentor intervention

Joint display combining qualitative and quantitative data into meta-inferences

Table 5 shows cardiac rehabilitation attendance. Most mentees (47%) were offered CR in the municipalities, and 71% attended at least one of the offered CR sessions (either physical training or dietary advice). A total of 53% were persistent and attended at least 50% of the physical training sessions, while 29% attended at least 50% of the dietary advice sessions. This is regarded as a high attendance rate, considering that a relatively large proportion of mentees did not have the opportunity to attend — 17% were not offered physical training sessions, and 65% were not offered dietary advice sessions. Qualitative data further expands on this by suggesting that the mentees rehabilitated themselves at home, in their own way. Furthermore, qualitative data illustrates the patient demand for rehabilitation sessions, indicating that patients are content with their own efforts to live a healthy life and feel no need for dietary sessions with professionals.

**Table 5:**
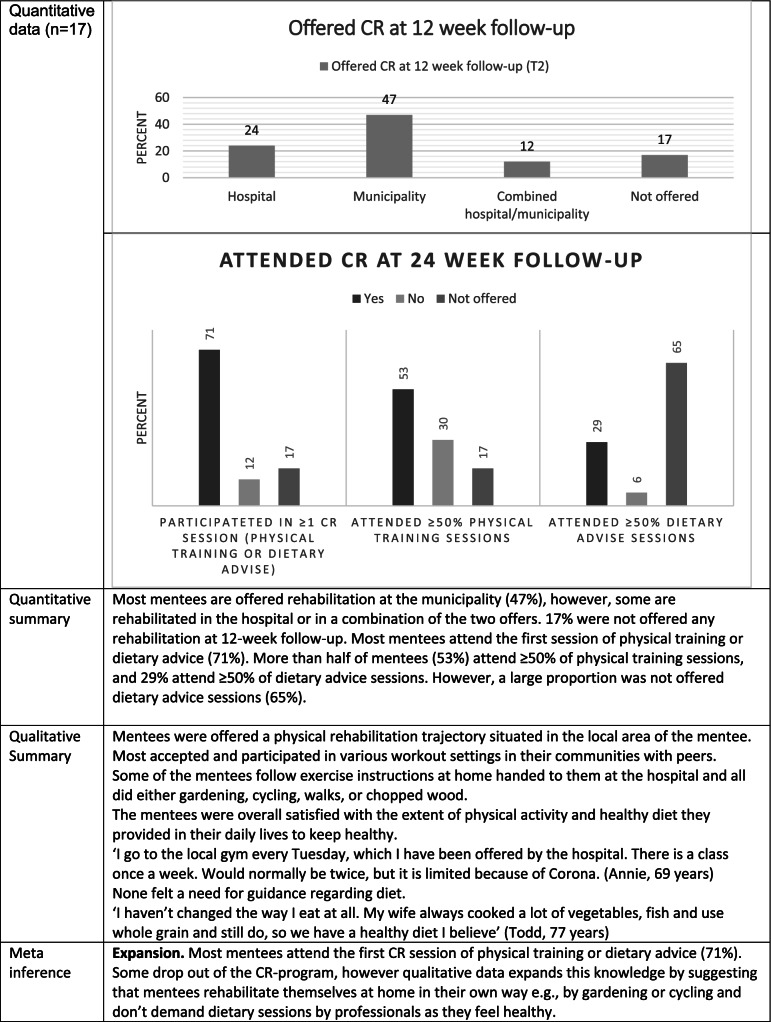
Joint display regarding cardiac rehabilitation attendance

*CR* cardiac rehabilitation. Joint display combining qualitative and quantitative data into meta-inferences

Table 6 displays changes in physical activity, diet, quality of life, symptoms of anxiety and depression, and self-efficacy among mentees at study follow-up, integrated with qualitative data. The quantitative and qualitative data reveal expanded, confirmatory, and discordant results.

**Table 6:** Joint display of changes in physical activity, dietary quality, quality of life, anxiety, depression, and self-efficacy

Variable	***N***	Level of variable	Baseline ***N***(%), median (Q1–Q3)	T1 N(%), median (Q1–Q3)	T2 ***N***(%), median (Q1–Q3)	Quantitative summary	Qualitative summary	Meta-inference
Physical activity > 30 min	17	0–1 times per week	5 (29)	2( 12)	2 (12)	No overall changes in physical activity. However, totally sedentary behavior (physical active 0–1 times per week) is reduced at follow-up	Most mentees work out in various workout settings in their communities *I have been offered and accepted a 3-month rehabilitation course at the gym where I go twice a week.* (Owen, 78 years) All the mentees recognize themselves as active in their daily lives compensating or supplementing the offered physical training sessions at CR. Some of the participants follow exercise instructions at home *I have been given a schedule of what exercises I should do from the hospital, so I have been working out at home with myself.* (Allan, 78 years)	**Expansion.** No overall increase in physical exercise could be detected. However qualitative data expands knowledge of mentee physical activities. As mentees practice a variety of psychical activities at the cardiac rehabilitation program or at home
		2–4 times per week	4 (24)	7 (41)	4 (24)			
		5–6 times per week	1 (6)	2 (12)	3 (17)			
		7–7+ times per week	7 (41)	6( 35)	8 (47)			
Dietary score	13	Fat score (0–100)	60 (47, 25–77, 25)	63 (50–77, 50)	70 (54,50–79, 59)	0% mentees practice a heart-healthy diet. And no overall improvement in heart healthy diet was observed. However, there was a 10-point increase in fat score	None of the mentees have changed their diet post MI. Some because they do not feel like going through the trouble of trying to eat differently *My daily life is the same. I eat the same as before. I haven’t changed anything... well, I do go for more walks now and exercise at a gym* (Allan, 78 years) Others believe they practice a healthy diet already eating vegetables, fish, and whole-grain and see no reason to optimize their diet accordingly *I haven’t changed anything in relation to diet or workouts. I have always eaten healthy and lived an active life* (Annie, 69 years)	**Discordance** Qualitative and quantitative findings are discordant. Qualitative data suggest that mentees regard themselves as practicing a healthy diet; however, quantitative data suggest that none of the mentees practice a heart-healthy diet
	15	Fish/fruit/vegetables score (0–100)	52 (45–61)	53 (42–59)	49.0 (45–58)			
Heart healthy diet	15	Yes	0 (0)	0 (0)	0 (0)			
		No	15 (100)	15 (100)	15 (100)			
Quality of life	15	HQol emotional (0–3)	2.25 (1.50–3.00)	2.75 (2.00–3.00)	3.00 (2.75–3.00)	Overall improvement in quality of life	Mentees live their life frontstage and view the heart disease as backstage. Living with a heart disease and the risk of potentially new cardiac events does not occupy the daily lives of mentees. Constrains, due to MI, would negatively impact the quality of life of the mentees and is therefore avoided *If we constrained ourselves all the time our quality of life would be very negatively impacted…we don’t want to go there. Maybe it would get you to be 95 but 90 is fine too* (Annie, 69 years) The mentees have their focus on living their lives without worrying about the future *My heart disease is backstage in my life…I don’t think about it that often* (Todd, 77 years)	**Expansion.** Quantitative findings indicate an improvement in quality of life. Qualitative findings expand this, by showing how mentees view the heart disease as backstage and life as frontstage, thus prioritizing their quality in life post MI, avoiding constraints that could limit quality of life
	15	HQol physical (0–3)	1.80 (0.90–2.29)	2.30 (1.70–2.90)	2.50 (1.80–3.00)			
	15	HQol global (0–3)	1.86 (0.93-2.36–	2.25 (1.79–2.86)	2.50 (2.07–3.00)			
Anxiety score	16	0–7, no anxiety	11 (69	14 (88)	13 (81)	31% of the mentees experience symptoms of anxiety and 6% symptoms of depression at baseline. All patients were free of depression symptoms at 24-week follow-up	Most mentees did not feel frightened or were anxious in the disease trajectory. They had a strong belief that everything was under control, and they were well-taking care of *I did not feel any fear… …I knew from the very beginning it was an MI because the paramedics told me, but it didn’t occur to me, that it could be dead serious before they attached the pads on my chest in the ambulance.* (Annie, 69 years) However, some did get worried at first, but these worries did not stay with them *I got a little scared at first because I have not tried anything like this before, but hey it’s probably just age telling you that you are an old bastard* (Todd, 77 years)	**Expansion** Interviews indicate that mentees are not that affected by their MI; however, quantitative findings indicate that some mentees do show signs of anxiety or depressive symptoms post MI; these symptoms are however diminished over time
		8–10, possible anxiety	2 (12)	1 (6)	2 (12)			
		11–21, proably anxiety	3 (19)	1 (6)	1 (6)			
	16	Anxiety median (Q1–Q3)	5.63 (2.00–8.75)	3.81 (1.00–6.50)	4.19 (1.25–6.00)			
Depression score	16	0–7 no depression	15 (94)	14 (88)	16 (100)			
		8–0, possible depression	1 (6)	2 (12)	0 (0)			
		11–21, probably depression	0 (0)	0 (0)	0 (0)			
	16	Depression median (Q1–Q3)	4.25 (2.25–7.00)	3.44 (1.00–4.75)	3.25 (1.25–5.75)			
Self-efficacy score	15	Self-efficacy median (Q1–Q3)	35 (30–38)	32 (30–38)	33 (30–39)	High levels of self-efficacy at baseline and follow-up	Overall, the mentees felt on top of the situation. They had different experiences to tap into, strengthening their coping abilities, e.g., previous serious illness or handling stressful events privately or at work *My husband also has a heart disease and has had several small strokes prior to my MI. So..we are familiar with severe diseases and know we are vulnerable in some way. We have had a lot of serious talks and experiences that we tap into in this new situation.* (Annie, 69 years) *I have a Master’s degree of Science (MSc) in Economics and Business administration, have received management training, done a myriad of courses, conferences, and seminars, and have had a lot of management jobs. I'm used to getting started on my own* (Sue, 80 years)	**Confirmation** Both qualitative and quantitative findings indicate that mentees possess high self-efficacy levels as mentees are used to having to deal with difficult situations in life and thus use life experience to strengthen self-efficacy

Joint display combining qualitative and quantitative data into meta-inferences. *N*, number. (%), percent. Q1–Q3, first-third quartile

No overall increase in physical exercise, measured quantitatively, was detected. However, totally sedentary behavior (physically active 0–1 times per week) was reduced at follow-up. The qualitative data expand on this by indicating that mentees practice a variety of psychical activities either at CR or at home.

No overall dietary changes were detected at follow-up, measured quantitatively. This is confirmed by qualitative data indicating that mentees made little effort to change their diet post-MI. However, there was a 10-point increase in fat score. All mentees were categorized as practicing a non-heart-healthy diet in quantitative diet screening, yet qualitative data shows a discrepancy. Qualitative data show that mentees define themselves as practicing a healthy diet, despite quantitative screening data indicating a non-heart-healthy diet.

Quality of life, measured quantitatively, increased during the follow-up period. This was the case for both emotional, physical, and global quality of life measures. Qualitative findings expand upon this by showing how mentees consider heart disease to be backstage, while life is frontstage — in other words, post-MI, they prioritize quality of life and deliberately avoid constraints that might limit this.

Qualitative data indicate that mentees are not that affected by their MI. However, quantitative findings expand upon this by showing that some mentees exhibited signs of anxiety (31%) or depressive symptoms (6%) post-MI. These symptoms diminished over time, and all patients were free of depression symptoms at 24-week follow-up.

Both quantitative and qualitative findings confirm that mentees possess high self-efficacy levels, as they are used to dealing with difficult situations in life. As such, mentees are able to draw on their life experience to strengthen self-efficacy.

## Discussion

To our knowledge, this is the first study to investigate the feasibility of a peer-mentor intervention in older vulnerable patients with MI in a cardiac-rehabilitation setting. The findings demonstrate that the peer-mentor intervention is feasible, acceptable for mentees, and safe for both mentees and peer mentors.

### Discussion of sample characteristics and recruitment capability

The mentees’ characteristics correspond to what would be expected in a MI population [3]. However, due to restrictive inclusion criteria, the inclusion rate was lower than expected. At first, the intention was that the study would only include older vulnerable women, but this proved a challenge in the clinical setting. As MI predominantly affects the male population, only a small proportion of the admitted patients were eligible, as there were additional inclusion criteria the women had to fulfill (≥ 65 years, lone dwelling, low socioeconomic position, or non-Western background). Focusing solely on the female MI population therefore presents practical challenges, and the peer-mentor intervention does not seem to be feasible in a population consisting solely of older vulnerable female patients with a poor state of health, as they lack interest and energy to engage in research and a peer-mentor intervention. As no strong scientific or clinical arguments were made for excluding the vulnerable male population, the inclusion criteria were broadened to include older vulnerable men.

The COVID-19 pandemic has had a major impact on the study, in relation to both the inclusion process and mentor-mentee contact. The study struggled to include peer mentors. This was partly explained by the fact that COVID-19 restrictions hampered the inclusion of peer mentors in the hospital setting. However, almost half of the eligible peer mentors (24 out of 47) declined to participate due to lack of time, energy, or interest. Being a volunteer peer mentor to another heart patient requires time, energy, and motivation. Our data indicate that the included mentors were both younger and more likely to have a higher education than mentees. Therefore, several inclusion areas might be considered to ensure a large eligible population of potential peer-mentors and enable the timely inclusion of peer-mentors in subsequent RCT studies.

### Discussion of acceptability and suitability of intervention and study procedures

The amount of peer-mentor support that mentees required (in terms of both time period and number of contacts) varied considerably. Most mentees needed eight mentor-mentee contacts, but there were variations — some needed 24-week support, others less. The variation in need might be due to variation in the patients’ cohabitation status and level of self-efficacy, as previous research has shown an association between patients’ support needs post-MI, self-efficacy levels, and perceived social support [29].

In our study, we were able to match all mentor-mentee couples in line with the mentees’ preferences, which might explain the high level of satisfaction and low dropout rate (15%).

The peer mentors did not make direct use of the two idea catalogues developed for the study. As such, idea catalogues are not deemed crucial for successful mentor-mentee meetings. However, they are still included in the study, as they might prove useful post-pandemic, when more face-to-face meetings might occur.

Only 11% of mentor-mentee contacts were face-to-face meetings. This is as expected, as peer mentors were instructed to comply with current COVID-19 restrictions. For the subsequent randomized controlled trial, we expect that more mentor-mentee meetings will be conducted face to face, depending on the COVID-19 situation, as the peer-mentors expressed a desire to meet their assigned mentee in person but abstained due to the pandemic.

Peer mentors were monitored to assess possible harm inflicted by the mentoring role. No indication of harm was detected during the mentoring period. This finding is in line with findings from similar peer-mentor projects, e.g., Nørskov et al. reported the mentor role to be safe, with no adverse events, in a population of severely ill patients with acute leukemia [8].

### Discussion of data-collection procedures and outcome measures

The mixed methods intervention design enabled us to achieve a comprehensive understanding of study feasibility and acceptability. The use of both quantitative and qualitative data revealed confirmatory, explanatory, and discordant results, which offered a better understanding of the complexity of the study endpoints that need to be captured in a future RCT.

Quantitative data regarding self-reported CR attendance is a valid method for collecting data regarding CR attendance [17]. This also proved to be an effective method among this older vulnerable population, as it ensured timely data collection with no missing values. Data collection via self-reported questionnaires at three time points (baseline, 12 and 24 weeks) had its difficulties among the population of older, vulnerable patients (mentees). Most attempts to ensure the timely and correct return of questionnaires (online and via mail) to facilitate the scheduled 12- and 24-week follow-up failed. As a consequence, the team relied on personally delivering questionnaires to most mentees. This helped to ensure the successful return of questionnaires, but it was time-consuming. As such, research budget should include time and travel expenses for personal delivery of questionnaires to be feasible in larger trials. Otherwise, the questionnaire was suitable and resulted in an acceptable number of missing values. Qualitative semi-structured interviews were conducted via telephone due to the COVID-19 situation. Although this method was regarded as safe and practical, it did hamper nonverbal communication. Face-to-face interviews will be preferred in future trials.

### Discussion of participant responses to intervention

The peer-mentor intervention shows signs of effectiveness. In our study, a relatively large proportion of the older vulnerable patients attended the first CR session (71%), and more than half attended ≥ 50% of the physical exercise program. This is a high attendance rate compared with findings in other vulnerable groups, in which, e.g., 25% of low-educated patients and 28% of older patients (> 69 years) attended the CR program [3]. A large proportion (65%) were not offered dietary advice sessions, and 17% was not offered a physical exercise program. This could be due to the relatively new organizational relocation of CR sessions from the hospital setting to a municipal setting in Denmark, possibly causing implementation difficulties and delays in CR offers in the municipalities. As such findings do indicate that the CR program needs to be optimized, so all patients in need of CR are offered CR. This calls for additional research as optimizing clinical CR was not the aim of the current study.

During follow-up, an increase in quality of life was observed among mentees, exceeding the minimal clinical difference [30]. In our study, the psychological measures of quality of life, as well as anxiety and depression, could have been influenced in a negative direction by the pandemic, as seen in other studies of cardiovascular patients [31]. It is therefore remarkable that an increase in quality of life among mentees was observed during the intervention and could indicate that peer mentors influenced the mentees’ psychological well-being during their disease trajectory. However, this needs to be further evaluated in the upcoming RCT study. The possible effect of peer mentoring on quality of life is plausible, as findings from another study show that peer-based education can increase both quality of life and self-care among younger (30–60 years) MI patients [32].

The study found that none of the mentees practiced a heart-healthy diet, either at baseline or at follow-up. This finding might have been influenced by the COVID-19 situation, as other studies indicate an overall increase in unhealthy lifestyle behaviors during the pandemic [31]. However, other studies initiated prior to the pandemic have shown similar results [33]. In Denmark, national guidelines recommend that MI patients are systematically screened for dietary intervention needs using the HeartDiet questionnaire [24]. As part of the CR program, patients categorized as not practicing a heart-healthy diet should be offered dietary advice. However, the study showed some discordance between the need for dietary advice and the dietary advice offered. Our data indicate that all mentees required dietary advice, as all were categorized as not practicing a heart-healthy diet. However, a large proportion (65%) were not offered dietary advice as part of the CR program. Previous research identified a gap between guidelines and clinical practice, as not all patients are systematically screened in the clinic for dietary intervention needs in the manner prescribed by the guidelines [24]. In our study, all patients were systematically screened, which revealed an additional gap between the need for dietary advice (those screened as not practicing a heart-healthy diet) and the dietary advice offered as part of the CR program.

### Strengths and limitations

The feasibility study’s trustworthiness was increased by investigator triangulation — at all steps of the analysis, multiple researchers discussed the findings to agree on the best interpretation of data. The quantitative questionnaire was composed of validated scales. The rigor of the qualitative data was ensured using Lincoln and Guba’s trustworthiness criteria, including dependability, credibility, confirmability, and transferability [34]. The second author (BB) conducted and coded all interviews and continuously discussed them with the first author (MP) throughout the analysis process to increase dependability and credibility. All authors participated in discussion of the final analysis, and the first and second authors (MP and BB) decided on the final themes. Field notes and a trail of initial interpretations and decisions ensured confirmability before the final themes were determined. A mixed methods intervention design was used to increase credibility and ensure a more comprehensive understanding of study endpoints by combining data from well-established quantitative and qualitative research methods [9].

We were not able successfully to include mentees or peer-mentors with a non-Western background. This has implications for the generalizability of the findings, as they might not apply to patients with a non-Western background. Additional research is warranted to clarify the feasibility of peer mentoring among this group of patients. However, previous research among similar populations does indicate that a very small proportion of cardiac patients are from non-Western/non-Danish-speaking countries [35].

## Conclusion

The peer-mentor intervention is feasible, acceptable for mentees, and safe for mentees and peer mentors. This paves the way for a full-scale peer-mentor intervention to be tested in a randomized controlled trial, with a view to decreasing inequality in cardiac rehabilitation attendance. However, some of the original study procedures were not feasible, and as such was revised. The mixed methods design enabled a broader understanding of data and revealed confirmatory, expanded, and discordant results. The peer-mentor intervention depends on volunteers, and a large eligible population is required in order to find motivated peer-mentors. The intervention shows signs of potentially positive effects in relation to increased cardiac rehabilitation attendance, as well as enhanced quality of life among older vulnerable MI patients. However, this needs to be further evaluated as part of a randomized controlled trial.

## Data Availability

The datasets used and/or analyzed during the current study are available from the corresponding author on reasonable request.

## Abbreviations

CR: Cardiac rehabilitation
MI: Myocardial infarction
PPI: Patient and public involvement
RCT: Randomized controlled trial
SEP: Socioeconomic position
